# Isolated Chronic and Transient Neutropenia

**DOI:** 10.7759/cureus.5616

**Published:** 2019-09-10

**Authors:** Navdeep Singh, Sandeep Singh Lubana, Lech Dabrowski

**Affiliations:** 1 Hospice and Palliative Care Medicine, North Shore Long Island Jewish Hospital, Brooklyn, USA; 2 Hematology and Medical Oncology, State University of New York Downstate Medical Center, Brooklyn, USA

**Keywords:** neutropenia, transient, reversible

## Abstract

Neutrophils are a critical part of the body’s defense system to prevent serious bacterial and fungal infections. Neutropenia is a term which is defined by the absolute neutrophil counts (ANC) < 1,500 cells/µL, and it becomes clinically significant when the level falls below 500 cells/µL. The risk of morbidity and mortality increases considerably when the levels fall below 200. In some ethnicities, the neutropenia is chronic and is frequently seen on routine outpatient visits. On the other hand, transient neutropenia is associated with a transient drop in the neutrophil count and many of the underlying causes are reversible. Patients and their families, as well as some clinicians, express great concern for neutropenia, leading to a multitude of tests and emergency room visits. In this review, we discuss the causes of both chronic and transient neutropenia. Also, we have given special emphasis on the mechanism of neutropenia and management of transient neutropenia.

## Introduction and background

Granulocytes include basophils, eosinophils, and neutrophils. These collectively are referred to white blood cells and neutrophils are the most abundant among all granulocytes. Neutrophils are an important part of the body’s defense system and prevent bacterial and fungal infections by phagocytosis and killing foreign invaders. A neutrophil count below 1,500 cells/μL is termed neutropenia, and it becomes clinically significant if the counts fall below 200, especially in immunocompromised states [1]. Hence, neutropenia is alarming to the patient families, as well as certain clinicians, even if it is transient. In this review, we have focused on the causes, mechanisms, and treatment of transient neutropenia. Transient neutropenia, in most circumstances, is reversible with removal of the offending agent. 

Neutropenia is defined as an absolute neutrophil count (ANC) < 1,500/μL. Depending on the severity of neutropenia, it may or may not impair the host immune defense system; however, it requires identifying the underlying cause. Once the count is < 500 cells/μL, it becomes clinically significant. If the level is < 200 (i.e., agranulocytosis), the risk of infection, including opportunistic infections, goes higher [1].

Benign ethnic neutropenia is a condition used to describe individuals of African descent with neutrophil counts less than 1.5 × 10^9^ cells/L in the absence of other causes. Benign ethnic neutropenia has also been reported in other ethnic groups, such as African-Caribbean people, West Indians, Ethiopians, and Yemenite Jews. It has been demonstrated that the patient with benign ethnic neutropenia has no increased risk of infection with normal bone marrow evaluation in terms of morphologic features and cellularity [2].

## Review

Transient neutropenia

The cause of transient neutropenia can be broadly categorized into infectious and noninfectious causes. Among infectious causes, viral infections are the most common cause [3-5]; however, bacterial and protozoal infections [6-7] are also associated with neutropenia. The non-infectious causes which cause transient neutropenia are further divided into inflammatory and autoimmune disease, as well as drug-induced. Most common viral agents include the Epstein-Barr virus, human immunodeficiency virus (HIV), influenza, parvovirus B19, and cytomegalovirus. Common bacterial causes include tuberculosis, brucella, paratyphoid and typhoid, and Anaplasma phagocytophilum. Among protozoan infections, Plasmodium vivax and P. falciparum are known to cause neutropenia [8].

Etiology and mechanisms of transient neutropenia

Neutropenia results either from the failure of production of neutrophils in the bone marrow or from their peripheral destruction. In 1931, Kracke first reported drug-induced neutropenia which was attributed to the use of an analgesic (pyramidon) in patients with agranulocytosis [9]. Due to polypharmacy, drug-induced neutropenia is most frequently seen in women and the elderly. Genetic and physiological traits may also play a role in the higher incidence. Recently, due to the increased use of chemotherapeutic agents, the incidence has greatly increased. The diagnosis of drug-induced neutropenia is suggested by the occurrence of febrile neutropenia with decreased signs of inflammation and reduction of ANC following drug exposure. Bone marrow biopsy reveals neutrophilic maturation arrest or hypercellularity with increased myeloid precursors and little maturation or myeloid hypocellularity [10].

Various mechanisms of drug-induced neutropenia are immune-mediated neutropenia, complement-mediated, dose-dependent inhibition of granulopoiesis, and direct toxicity for myeloid precursors.

Immune-mediated neutropenia is rapid in onset, usually a few hours to a few days, especially if there is prior exposure to the drug [11]. Certain drugs act as haptens, which causes antibody formation against the neutrophils and results in their destruction. For example, penicillin has been shown to act as a hapten, inducing immune neutropenia in patients receiving high doses of cephalosporins, with the low neutrophil counts returning to normal after ceasing drug administration [12].

Another mechanism of immune-mediated neutropenia is apoptosis. The life span of neutrophils in circulation is eight to 20 hours following which they undergo apoptosis. Some drugs, e.g., clozapine, hasten the apoptosis process which has been demonstrated by in vitro studies. Clozapine is metabolized by P450 and peroxidase to an unstable toxic metabolite, leading to depletion of intracellular glutathione (GSH) and resulting in neutrophil destruction. GSH is crucial for the detoxification of H_2_O_2_ that has diffused into the cytosol [13]. 

The antithyroid medications, i.e. propylthiouracil, are associated with the formation of an antineutrophil cytoplasmic antibody (ANCA) and causes neutropenia via the complement-mediated mechanism [14].

Dose-dependent inhibition of granulopoiesis is seen by drugs, such as beta-lactam antibiotics [15], carbamazepine [16], and valproic acid [17]. These drugs give variable results at low concentrations. However, at higher concentrations, these drugs inhibit colony-forming units of granulocytes and macrophages [15-17].

There are many drugs that can cause direct toxicity for myeloid precursors resulting in transient neutropenia. These include chemotherapy medications melphalan, busulfan, methotrexate, carboplatin, cisplatin, cis-diammine, dichloroplatinum, paclitaxel, cyclophosphamide, doxorubicin, etoposide, and vinorelbine [18].

Chronic neutropenia

Chronic autoimmune neutropenia and chronic benign (or idiopathic) neutropenia in children have excellent outcomes and are difficult to distinguish diagnostically. Hence, these terms are nearly interchangeable. The usual age of presentation is during infancy with normalization of the neutrophil count by the age of two to four years [1]. This form of neutropenia is very rare with an incidence of about one in 100,000 children/year [19]. Chronic autoimmune/benign/idiopathic neutropenia of childhood shares many clinical, laboratory, therapeutic, and prognostic features. The median age of diagnosis is eight to 11 months, patients usually get minor infections (with upper respiratory infections being the most common among them), and rarely, gingivitis. Serious infections are rare and are usually seen in young infants. The median ANC count at the time of diagnosis is around 200 cells/μL with a normal hemoglobin and platelet count. Bone marrow biopsy would show normal to increased myelopoiesis. Recurrent otitis media may require the use of prophylactic antibiotics. Patients with acute bacterial infections are managed with antibiotics. Granulocyte-colony stimulating factor (G-CSF) is only recommended in the case of serious infections. G-CSF can also be used to prevent frequent emergency department visits or admission in order to improve the quality of life of patients. Prognosis is excellent in most patients [20].

At the time of the bacterial infection or stress, the neutrophil count generally rises, which is an important diagnostic indicator that the bone marrow storage pool of neutrophils are adequate in number. It also rules out hypoproductive defects, such as severe congenital neutropenia. Administration of glucocorticoids will produce the same effects [21-22].

Chronic autoimmune and idiopathic neutropenia in adults cannot be differentiated due to the lack of reliable antineutrophil antibody tests. It is more common in women compared to men. Also, in women, it is more likely to be chronic when compared to men [8]. Recurrent stomatitis and skin or respiratory infections can be seen during the period of very low ANCs but most patients will develop severe infections [1].

Large granular lymphocytic (LGL) leukemia mostly presents with isolated chronic neutropenia compared to other forms of leukemia where other cell lines are also lost [23].

Systemic lupus erythematosus, rheumatoid arthritis, Sjogren syndrome, sarcoidosis, and other chronic inflammatory and autoimmune diseases are often the cause of mild and intermittent neutropenia [24].

Diagnosis and management

Neutropenia could sometimes be secondary to laboratory error and should be confirmed by repeating the blood test. History and laboratory evaluation can help identify some of the causes of transient neutropenia. The antineutrophil antibody is unreliable for the diagnosis of chronic neutropenia due to high false-negative and false-positive rates. However, the detection of antibodies against specific epitopes is more reliable [25]. Identification of a specific antibody in maternal serum in case of neonatal isoimmune neutropenia can confirm its diagnosis [26].

Neutropenia can be a sign of underlying immunodeficiency or systemic disease (autoimmune disorder and leukemia or myelodysplastic disorder). To evaluate immunodeficiency, immunoglobulin levels as a screening tool should be performed. In the case of systemic disorders, such as leukemia or myelodysplastic disorders, a bone marrow biopsy is indicated. For systemic autoimmune diseases, the signs and symptoms of the autoimmune disorder should be thoroughly investigated [27-29].

Adequate ANC rise in response to infection, or the demonstration of a twofold or greater rise in ANC following glucocorticoid challenge, rule out congenital neutropenia. A single dose of prednisone (1 - 2 mg/kg) leads to mobilization of the bone marrow reserve pool of mature neutrophils to the periphery. Blood testing for ANC determination should be performed before and four to six hours following steroid administration. Next-generation sequencing testing is a non-invasive test that has been in use for congenital neutropenia or bone marrow failure [21-22, 30].

Clinical features suggestive of myelodysplastic syndromes or leukemia requires rapid diagnosis by bone marrow examination [1].

Neutropenia is managed depending on the underlying cause. Transient neutropenia, for most cases, tends to resolve spontaneously without any clinical sequela. Also, managing the underlying cause of transient neutropenia resolves it. The drug-induced neutropenia usually resolves following discontinuation of the offending drug. However, in the case of polypharmacy, it may be hard to identify the offending agent. There are certain circumstances where the discontinuation of a particular medication is not feasible and requires to be continued despite the neutropenia. One such example is clozapine used for the treatment of schizophrenia. Clozapine can be continued if the patient has an adequate number of neutrophils, usually 500 - 1,000 cells/μL. It is important to evaluate the relative risk of severe agranulocytosis versus discontinuation of clozapine (leading to high mortality due to inadequate treatment for schizophrenia). Caution must be used and regular monitoring of the neutrophil count should be performed if the drug is restarted [31-32].

## Conclusions

In this review, we have highlighted the importance of recognizing the reversible causes of neutropenia by careful history taking and keeping in mind the temporal relationship of the time of starting the offending agent or underlying viral infections leading to transient neutropenia. This could alleviate anxiety and fear of the patient and their families and would also reduce the financial burdens of treatment significantly. 
